# Depression of the soil arbuscular mycorrhizal fungal community by the canopy gaps in a Japanese cedar (*Cryptomeria japonica*) plantation on Lushan Mountain, subtropical China

**DOI:** 10.7717/peerj.10905

**Published:** 2021-03-15

**Authors:** Guiwu Zou, Yuanqiu Liu, Fanqian Kong, Liqin Liao, Guanghua Deng, Xueru Jiang, Junhuo Cai, Wei Liu

**Affiliations:** 1 Jiangxi Provincial Key Laboratory of Silviculture, Collaborative Innovation Center of Jiangxi Typical Trees Cultivation and Utilization, College of Forestry/College of Art and Landscape, Jiangxi Agricultural University, Nanchang, Jiangxi, China; 2 Positioning Observation Station of Forest Ecosystem in Lushan, Lushan National Nature Reserve of Jiangxi, Jiujiang, Jiangxi, China; 3 College of Life Sciences, Zhejiang University, Hangzhou, Zhejiang, China

**Keywords:** AMF, Canopy gaps, Symbioses, Succession

## Abstract

Both canopy gaps (CG) and arbuscular mycorrhizal fungi (AMF) play key roles in seedling establishment and increasing species diversity in forests. The response of AMF to canopy gaps is poorly understood. To assess the long-term effects of canopy gaps on soil AMF community, we sampled soil from plots in a 50-year *Cryptomeria japonica* (L.f.) D. Don. plantation, located in Lushan Mountain, subtropical China. We analyzed the AMF community, identified through 454 pyrosequencing, in soil and edaphic characteristics. Both richness and diversity of AMF in CG decreased significantly compared to the closed canopy (CC). The differences of the AMF community composition between CG and CC was also significant. The sharp response of the AMF community appears to be largely driven by vegetation transformation. Soil nutrient content also influenced some taxa, e.g., the low availability of phosphorus increased the abundance of *Acaulospora*. These results demonstrated that the formation of canopy gaps can depress AMF richness and alter the AMF community, which supported the plant investment hypothesis and accentuated the vital role of AMF–plant symbioses in forest management.

## Introduction

Forests play a critical role in global carbon (C) sequestration (Curtis et al., 2018), harboring most of the biodiversity and sustaining the economy (Gardner et al., 2009). When one or a few trees die (or are injured) in the forest canopy, whether caused by natural or artificial disturbances, it generates small openings, which we call ‘gaps’ (Hubbell et al., 1999). As one of the most dominant results of disturbances, canopy gaps play an important role in driving forest ecosystem succession and regeneration and maintaining biodiversity (Chen et al., 2019; Muscolo et al., 2014). Directly or indirectly, canopy gaps greatly enhance the environmental heterogeneity, such as higher soil water content (Wang et al., 2018), higher soil and air temperature, and stronger respiration in canopy gaps (Muscolo et al., 2014). Moreover, canopy gaps can induce the variation of soil nitrogen (Schliemann & Bockheim, 2014) and microbial biomass (Lewandowski et al., 2019), and the sharp decrease of ectomycorrhizal fungi richness (De Groot et al., 2016). Forest scientists and ecologists have been attracted to gaps dynamics, because the gaps dynamics are closely associated with practical, foreseen applications (e.g., forest conservation practice, the natural regeneration method), as well as basic ecological theories (e.g., niche partitioning, species adaptation, latitudinal gradient of species diversity) (Brokaw & Busing, 2000; Yamamoto, 2000; Lewandowski et al., 2019).

Arbuscular mycorrhizal fungi (AMF) are members of the Glomeromycota, which form the most common and widespread terrestrial plant symbioses (Smith & Read, 2008), and are believed to support plant growth by increasing the supply of immobile soil nutrients, notably P, enhancing tolerances or resistance to soil pathogens and abiotic stresses, and by improving the soil structure (Konvalinková, 2017; Hazard & Johnson, 2018). Meanwhile, AMF benefit edaphic health by decreasing the abundances of soil-borne pathogenic fungi (Liu et al., 2020). The hypothesis that AMF diversity is crucial for maintaining productivity and stability of ecosystems has now been put on a firm footing by experimental observations (Van der Heijden, Bardgett & Van Straalen, 2008), which showed that increasing AMF species richness led to an alteration in the plant community structure and an increase in plant productivity and diversity (Bennett et al., 2017). Because of their pivotal role in plant community ecology and multiple beneficial effects on plants as well as on edaphic health, AMF can be beneficial in revegetation and restoration of degraded soil and forests (Maltz & Treseder, 2015). Moreover, ecologists have recognized the important role of AMF in the succession and stabilization of plant community structure (Bennett et al., 2017; Koziol & Bever, 2015).

Few studies reported forest gaps reduced endomycorrhizal biomass (Schliemann & Bockheim, 2014) and AMF abundance in soil microbial community (Lewandowski et al., 2015) however, their resolution is low due to the method limitation. Whether and what kind of effects canopy gaps will have on AMF communities is still little understood. This inevitably hampers our understanding of the response of the AMF community to canopy gaps. To unravel the effects of canopy gaps on AMF community in higher resolution, we applied 454 sequencing to track the richness, diversity, community composition of AMF in the soil of Japanese cedar canopy gaps and the soil under the canopy. In parallel, we detected the physicochemical properties of soil to analyze the relationships between soil characteristics and AMF communities. We expected that (1) the richness and diversity of AMF are higher in canopy gaps (CG) than that in closed canopy (CC) since the long-term effects of the canopy gaps, (2) the assemblage of the AMF community would differ significantly between the canopy gaps and closed canopy.

## Materials & Methods

### Study site description

The study was conducted at the Lushan Nature Reserve (29°26′–29°41′N, 115°52′–116°08′E), south part of Jiujiang City, Jiangxi Province, China. Lushan is an isolated mountain located in the center of the vast plain of the middle and lower reaches of the Yangtze River, and northwest of Poyang Lake. The region has a subtropical humid monsoon climate, and the mean annual temperature is 11.4 °C (the extreme maximum temperature was 32.8 °C, and the extreme minimum temperature was −16.8 °C). The mean annual precipitation is 1929.2 mm, which is about 500 mm higher than that in the plain area on the same latitude, with 70% of the precipitation concentrated in the period from April to July (Liu & Wang, 2010).

Lushan has complex landscapes containing block mountain tectonic landforms, fluvial landforms and glacial topography (Liu & Wang, 2010), which cover an area of about 300 km^2^ with an altitude range from 30 to 1 474 m. The soil type in our study site is yellow-brown soil (Wang et al., 2010). Vegetation is evergreen forest dominated by several Fagaceae trees including *Castanopsis sclerophylla* Lindl., *C. eyrie* Champ. and *Lithocarpus glaber* Thunb., as well as some evergreen woodland species at low altitudes (50-600 m). Deciduous trees grow at middle altitudes (600–1,000 m), where some *Japanese cedar* monocultural plantations were introduced about 60 years ago (Liu & Wang, 2010).

Japanese cedar, which originated in Japan, has been widely introduced in China, due to its high ornamental, economic and ecological value. It has been known for years that Japanese cedar roots can be colonized by mycorrhizal fungal species (Yamato & Iwasaki, 2002). The diameter of the introduced Japanese cedar in Lushan Mountain is up to 50 cm at breast height now. The planted seedlings were 2–3 years old, generally about 70 cm in height and 0.6–1.0 cm in ground diameter. Managed twice a year for five years after afforestation to maintain them as monospecific stand. Management practice is mowing grass and shrubs in summer, then mowing again and losing surface soil in September. Plant residues from tending were covered around *C. japonica*. To improve the light conditions and cultivate large diameter timber, trees with poor growth, pests and diseases, and broken shoots were thinned twice during 5 to 8 years and 10 to 15 years after afforestation (Ju & Xu, 1986).

### Experimental design and sampling

Three canopy gaps (CG, about 20 m × 20 m) and closed canopy (CC) (i.e., different habitats) were chosen as study plots, based on a comprehensive investigation of topography, density, tree diameter and canopy gaps size. The fieldwork was conducted according to “Observation Methodology for Long-term Forest Ecosystem Research” of National Standards of the People’s Republic of China (GB/T33027-2016). The gaps, formed by the forest form transformation (i.e., transforming forest structure artificially) in 2012, have nurtured some herbs and shrubs at the time of sampling. We established a quadrate (10 m × 10 m) in the center of each canopy gaps, and the distance from the quadrate edge to the projection of neighbor *C. japonica* crown is about 3–5 m. To ensure the consistency of potential confounding factors (e.g., slope, aspect, tree age), we chose the plots at a small distance from each other. There are few *Lophatherum gracile* Brongn. scattered under the closed canopy of *C. japonica*, whereas more plant species scattered sparsely in the canopy gaps, they are *Lindera erythrocarpa* Makino., *Symplocos stellaris* Brand., *Polygonatum sibiricum* Delar. ex Redoute., *Rubus corchorifolius* L.f., *Macleaya cordata* (Willd.) R. Br., *Aster ageratoides* Turcz., *L. gracile*, and some unidentified gramineous. More information about the plots is available in the table below (Table 1).

**Table 1:** Basic information of the plots.

**Habitat**	**Stand density**	**Canopy density**	**Overstory**	**Understory**
Canopy gap	7783 ±195 plants hm^−1^	0	No	Over 8 species
Closed canopy	863 ±105 stems hm^−1^	0.8	*Cryptomeria japonica*	1 species

Soil samples at each plot were collected randomly and homogeneously from nine points using a 6 cm-diameter stainless-steel earth borer in November 2014. Previous studies have shown that AM hyphae, total fungal colonization was significantly different between the 0–10 cm soil layer and >10 cm soil layer (Yang et al., 2010), so we sampled the soil of 0–10 cm and 10–20 cm respectively. To strengthen the representativeness of samples, soils from the nine points of the same layer were mixed into one sample, with 6 soil samples (3 in 0–10 cm and 3 in 10–20 cm) per habitat. Afterward, each soil sample was mixed and homogenized by passing through <2 mm sieve to remove above-ground plant materials, roots and stones, then divided into two subsamples. One subsample was stored at −80 °C for DNA extraction and subsequent molecular analysis and the other was air-dried, gently ground and passed through a 0.149 mm mesh sieve for future chemical analyses.

### Soil chemical property determination

Soil moisture content was determined by a drying method (at 105 °C, for 6 h). Soil pH was measured in H_2_O with a soil-to-solution ratio of 1:5. Soil organic matter (SOM) concentration was measured using the dichromate oxidation method (Kalembasa & Jenkinson, 1973). Total nitrogen (TN) was estimated by Kjeldahl digestion (Lu, 2000). Soil total phosphorus (TP) was determined by the molybdenum blue colorimetric method after oxidation by sulfuric and perchloric acid (Lu, 2000). Soil available phosphorus (AP) was extracted with 0.5 mol L^−1^ sodium bicarbonate (NaHCO_3_) and determined in the same way as that of total phosphorus. Available potassium (AK) was extracted by 1 mol L^−1^ ammonium acetate (CH_3_COONH_4_) and then detected by a flame spectrophotometer (Lu, 2000).

### DNA extraction, PCR amplifications and molecular identification of AM fungi

Genomic DNA was extracted from 0.5 g fresh soil by using a FastDNA SPIN Kit for Soil (Aidlab Biotechnologies Co., Ltd) following the manufacturer’s instructions. The extracted soil DNA was diluted to 10-20 ng µL-1 by double-distilled H_2_O and stored at −20 °C until added into a nested PCR with AML1/AML2 (Sequences 5′–3′ are ATCAACTTTCGATGGTAGGATAGA/ATCAACTTTCGATGGTAGGATAGA) (Lee, Lee & Young, 2008) and NS31/AM1 (Sequences 5′- 3′ are TTGGAGGGCAAGTCTGGTGCC/GTTTCCCGTAAGGCGCCGAA) (Simon, Lalonde & Bruns, 1992; Helgason et al., 1998). The latter primer pair was augmented with the 454 pyrosequencing adapters and 7 bp-long barcodes for multiplexing (Xiang et al., 2014).

The first PCR (10 µL volume) contained 5 µL 2 × Mix, 0.4 µL primer (AML1 and AML2, 10 µmol L^−1^, 0.2 µL each; concentration in final volume is 200 nmol L^−1^), 0.12 µL BSA, 3.48 µL ddH_2_O and 1 µL DNA template with the following cycling conditions: (1) 94 °C for 3 min, (2) 94 °C for 30 s, (3) 58 °C for 45 s, (4) 72 °C for 45 s, (5) 72 °C for 10 min; 30 cycles from (2) to (4). The second PCR (50 µL volume) consisted of 25 µL 2 × Mix, 1.6 µL primer (NS31 and AM1, 10 µmol L^−1^, 0.8 µL each; concentration in final volume is 160 nmol L^−1^), 21.4 µL ddH_2_O and 2 µL DNA template, which was diluted with double-distilled H_2_O (1: 10) from the first amplification product, and conducted under the same conditions as in the original PCR except the time of (3) changed to 30 s and the number of cycles performed (32 instead of 30). The last PCR products were separated by 2% agarose gel (120 V for 40 min) and purified with an extraction kit (Aidlab Biotechnologies Co., Ltd) according to the manufacturer’s instructions, then quantified using *QuantiFluor™*-*ST* (*Promega*, USA). As a method of high throughput DNA sequencing, 454 pyrosequencing provided a powerful approach to assess AMF diversity (Öpik et al., 2009) and has been used in a grassland ecosystem to shed more light on the AMF community (Xiang et al., 2014). The quantified amplicons were sequenced on a 454-PLX+ system at the LS454 platform (Shanghai, China). Each sample provided an average of 10,000 sequencing reads. The availed sequences (including the barcode and primer sequences, except for ambiguous nucleotides) were enough for analysis. Unique sequences were clustered into operational taxonomic units (OTUs) using the unsupervised Bayesian clustering algorithm CROP with a 97% identity threshold, and the most abundant sequence from each OTU was selected as a representative sequence for that OTU. The cluster of sequences was performed by Usearch (version7.1 http://drive5.com/uparse/). The UNITE (version.6.0 http://unite.ut.ee/index.php) served as the reference database for fungal taxonomy (Koljalg et al., 2013). The representative sequences were further confirmed by Genbank (http://www.ncbi.nlm.nih.gov/) for the OTUs that could not be identified at the level of families or classes in the above fungal database.

### Statistical analyses

We used analysis of variance (ANOVA) to test the differences of soil properties, AMF richness and diversity, and the number of individuals per OTU between CC and CG. Least significant difference (LSD) was used for multiple comparisons of differences between samples. All these analyses were carried out using R version 3.6.0 (R Core Team, 2019). The figures were drawn by SigmaPlot (14.0).

Principal component analysis and canonical correspondences analysis based on the abundance of OTUs were performed by CANOCO 4.5. To demonstrate the statistical differences of AMF community composition between CC and CG, PERMANOVA was carried out with the *vegan* package (Oksanen et al., 2019) in R. Differences at *p* < 0.05 was considered statistically significant. We use asterisks (*) to indicate *p* < 0.05, ** for *p* < 0.01 and ns (no significant) for *p* > 0.05 in all the figures and tables of this paper.

## Results

### Physical and chemical properties of soil

The upper layer soil of closed canopy (CC) had significantly higher soil moisture content (*p* < 0.01) and pH value (*p* < 0.05) compared to the other soil (Table 2). In the canopy gaps (CG) the available K content in the upper layer (0–10 cm) was significantly higher than that of the other soil samples (*p* < 0.01, Table 2). Total nitrogen content in the 0-10-cm soil layer was significantly higher than in the 10–20 cm soil layer in both habitats (*p* < 0.05). All the soil properties—except for the available K content ¬—were higher in CC, although the changes were not significant. Meanwhile, the value of all soil characteristics—except for total P ¬—showed a decreasing tendency as the soil depth increased.

**Table 2:** Characteristics of soil (mean ± SE, *n* = 3) in different habitats and depths.

**Habitat**	**Soil depth**	**Soil water content (%)**	**SOM (g kg^−1^)**	**TN (g kg^−1^)**	**TP (mg kg^−1^)**	**AP (mg kg^−1^)**	**AK (mg kg^−1^)**	**pH value**
CC	0–10 cm	86.53 ± 6.67^a^	117.2 ± 16.0^a^	4.55 ± 0.6^a^	424.4 ± 52.8^a^	67.9 ± 24.4^a^	83.1 ± 8.8^b^	4.70 ± 0.12^a^
	10–20 cm	58.43 ± 0.75^b^	81.0 ± 11.3^a^	2.80 ± 0.4^b^	550.3 ± 67^a^	37.5 ± 8.4^a^	65.9 ± 8.8^b^	4.43 ± 0.02^b^
CG	0–10 cm	59.97 ± 0.67^ab^	93.3 ± 12.4^a^	4.33 ± 0.2^a^	391.1 ± 84.2^a^	32.4 ± 2.0^a^	125.2 ± 12.5^a^	4.40 ± 0.06^b^
	10–20 cm	57.17 ± 2.14^b^	83.4 ± 10.2^a^	2.76 ± 0.1^b^	508.1 ± 106.8^a^	41.2 ± 16.4^a^	67.8 ± 11.6^b^	4.57 ± 0.02^ab^
Dependence of soil characteristics on habitat, soil depth, and their interactions in ANOVAs (*F* value)								
Habitat		63.67^***^	0.72	0.11	0.22	1.08	4.34	1.26
Depth		50.69^**^	3.29^*^	18.87^**^	2.29	0.5	12.47^**^	0.42
Habitat*Depth		55.87^*^	1.07	0.51	0.003	1.63	3.62	10.07^*^

**Notes.**

CC Closed Canopy CG Canopy Gap SOM soil organic matter TN total nitrogen TP total phosphorus AP available phosphorus AK available potassium

Means with the same letters are not significantly different (a one-way ANOVA, the LSD test, at the 5% level).

* *p* < 0.05.

** *p* < 0.01.

*** *p* < 0.001.

Significant results (*p* < 0.05) are shown in bold.

### Overall pyrosequencing information

**Figure A1:**
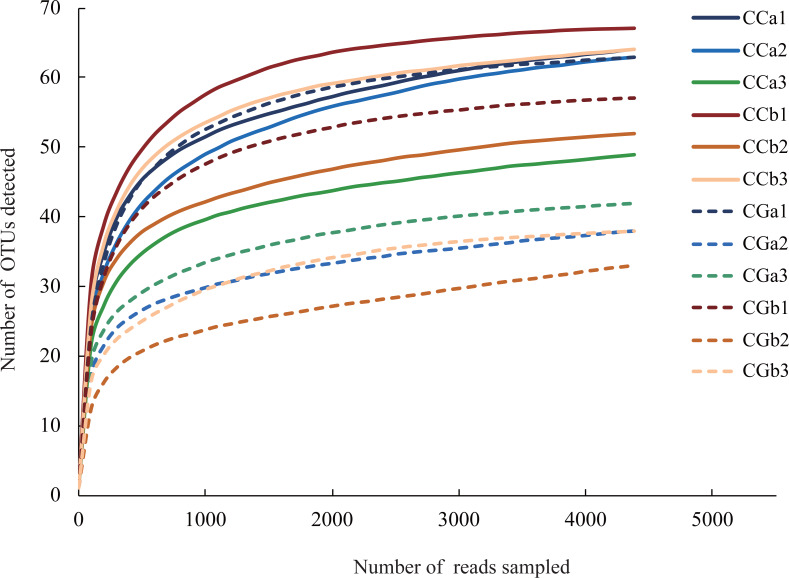
The rarefaction curve of OTUs.

A total of 40 471 trimed sequences (ranging from 4 724 to 8 830 reads per sample) with a length >200 bp were obtained from the CC and 45 140 (ranging from 5 781 to 9 218 reads per sample) from the CG. The length of trimed sequence is mostly between 201–500 bp. Rarefaction curves for the AMF community indicated that our sequencing depth has reached the threshold and can detect the vast majority of OTUs (Fig. A1).

### The richness and diversity of AMF

We detected a total of 91 OTUs in all the samples and 85.71% (78 OTUs) of which existed in both CC and CG (Appendix S1, Table SA1). In more detail, 58.5 OTUs per sample were detected in CC, which was significantly higher than the 44.66 in CG (*p* < 0.05). In general, the richness (numbers of OTUs), number of estimated asymptotic AMF taxon richness (Chao index, *p* < 0.01), Evenness (*p* < 0.05) and Shannon index (*p* < 0.01) in the CC were significantly higher than those in the CG. All indexes in CC showed an increasing trend with increasing soil depth, and this tendency was opposite to that observed in CG, although the changes were not significant (*p* > 0.05) (Table 3).

**Table 3:** The richness and diversity of AMF (mean ± SE, *n* = 3) in different habitats and depths.

**Habitat**	**Soil depth**	**Number of OTUs**	**Shannon index**	**Simpson index**	**Eveness index**	**Chao index**
CC	0–10 cm	57.00 ± 5.03^a^	2.96 ± 0.09^a^	0.9199 ± 0.007^a^	0.7154 ± 0.024^a^	59.37 ± 5.10^a^
	10–20 cm	60.00 ± 4.58^a^	3.02 ± 0.13^a^	0.9256 ± 0.007^a^	0.7392 ± 0.020^a^	62.57 ± 4.56^a^
CG	0–10 cm	45.67 ± 7.17^a^	2.39 ± 0.10^ab^	0.8297 ± 0.017^a^	0.6294 ± 0.004^ab^	48.14 ± 6.28^ab^
	10–20 cm	41.66 ± 7.31^a^	2.02 ± 0.43^b^	0.7099 ± 0.137^a^	0.5379 ± 0.096^b^	42.39 ± 2.36^b^
Dependence of richness and diversity indexes of AMF on habitat, soil depth, and their interactions in ANOVAs (*F* value)						
Habitat		5.82^*^	11.29^**^	4.87	8.04^*^	10.74^*^
Depth		0.01	0.43	0.68	0.45	0.07
Habitat*Depth		0.32	0.90	0.82	1.3	0.87

**Notes.**

CC Closed Canopy CG Canopy Gap

Means with the same letters are not significantly different (a one-way ANOVA, the LSD test, at the 5% level).

* *p* < 0.05.

** *p* < 0.01.

Significant results (*p* < 0.05) are shown in bold.

### Composition of AMF communities

**Figure 1:**
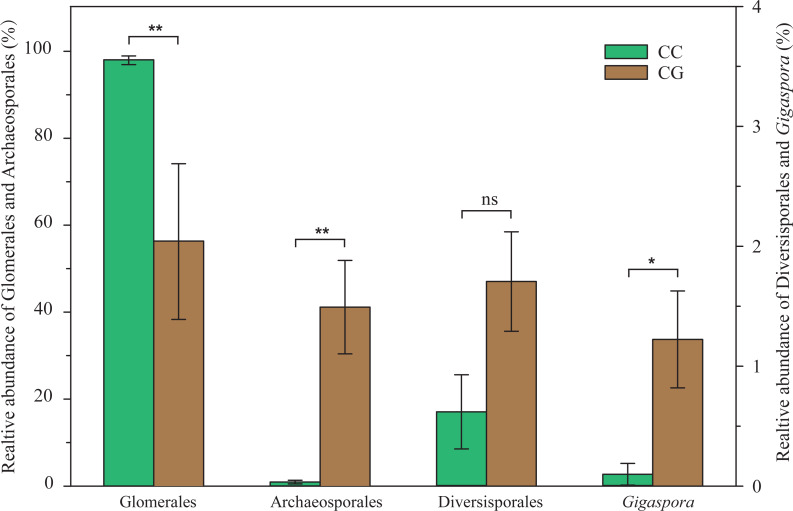
The relative abundance of main AMF orders and genera in different habitats. Data are mean ± se (*n* = 6). CC, Closed Canopy; CG, Canopy Gap. ns, no significant; *, *p* < 0.05; **, *p* < 0.01.

**Figure A2:**
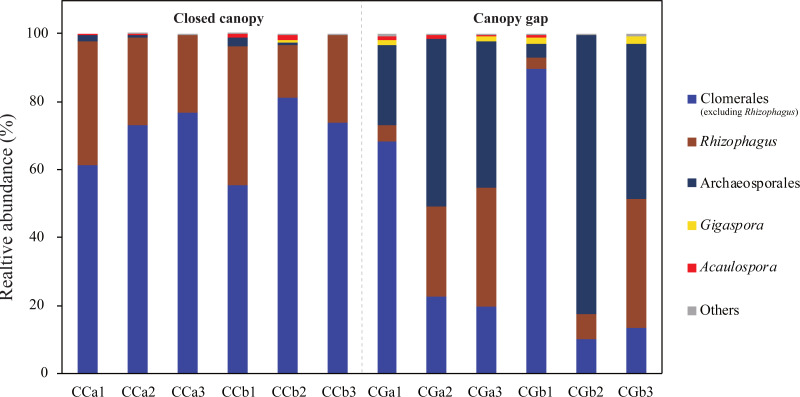
The detailed relative abundance of all groups. CCa and CCb indicate 0–10 cm and 10–20 cm soil in closed canopy (CC) respectively, CGa and CGb for 0–10 cm and 10–20 cm soil in canopy gap (CG) respectively. The numeral 1, 2 and 3 mean different plots within the same treatment (three repetitions per treatment).

All the sequences came from the orders Glomerales, Archaeosporales and Diversisporales (containing genera *Acaulospora* and *Gigaspora* here) except the few unclassified sequences. In the CC habitat, Glomerales had an absolute advantage (98.16%), followed by Archaeosporales (1.03%) and Diversisporales (0.06%). In the CG habitat, although Glomerales (56.47%) was still the most dominant group, the relative abundance of Archaeosporales increased dramatically to 41.25%, and Diversisporales also increased to 1.71%. Compared to the CC, the relative abundance of Glomerales decreased significantly (*p* < 0.01) and Archaeosporales increased significantly (*p* < 0.01) in CG (Fig. 1). At the genus level, the relative abundance of *Rhizophagus* did not differ significantly in the two habitats (27.28% in CC, 19.17% in CG, *p* > 0.05). Although the increment of Diversisporales was not significant (*p* > 0.05), the relative abundance of *Gigaspora* (1.22%) in the CG increased significantly (*p* < 0.01) compared to almost none in CC. The relative abundance of various groups did not change significantly with the increase of soil depth, nor did it show a regular trend. The detailed taxonomic distribution based on sequences obtained in soil samples is presented in the Fig. A2.

Although 87 OTUs appeared in both habitats, 20 of them fluctuated in abundance significantly between habitats. In addition, compared to the CC, 8 OTUs were not observed and 5 new OTUs were observed in CG (Appendix S1, Table SA1).

**Figure 2:**
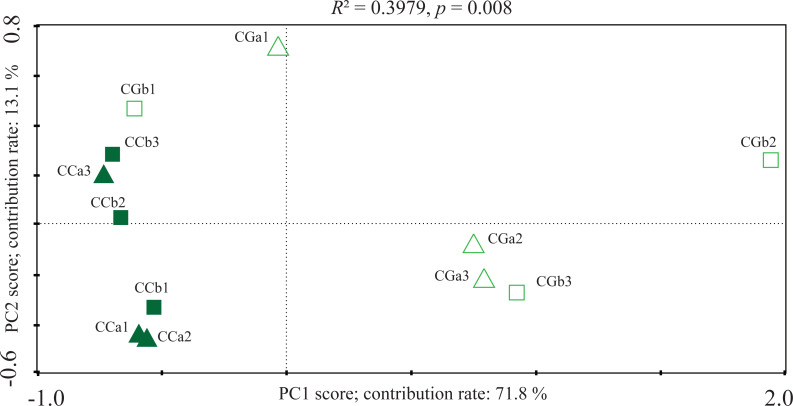
Principal component analyses (PCA) of AMF community in different habitats and layers based on OTUs abundance. CCa and CCb indicate 0–10 cm and 10–20 cm soil in closed canopy (CC) respectively, CGa and CGb for 0–10 cm and 10–20 cm soil in canopy gap (CG) respectively. The numeral 1, 2 and 3 mean different plots within the same treatment (three repetitions per treatment).

**Figure 3:**
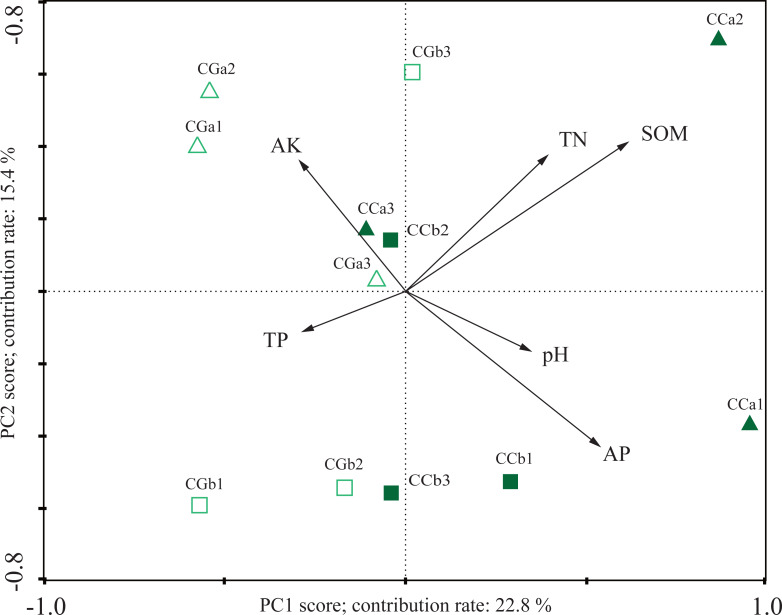
The canonical correspondence Analysis (CCA) of AMF community composition and soil properties. CCa and CCb indicate 0–10 cm and 10–20 cm soil in closed canopy (CC) respectively, CGa and CGb for 0–10 cm and 10–20 cm soil in canopy gap (CG) respectively. The numeral 1, 2 and 3 mean different plots within the same treatment (three repetitions per treatment).

Simultaneously, principal component analysis (PCA) based on OTUs abundance displayed the difference in the AMF community between CC and CG (Fig. 2). The first principal component mainly distinguished the AMF community in the CG from that of the CC (contributed 71.8%), which suggested a strong fluctuation of the AMF community. The distinctions were verified by PERMANOVA (*R*^2^ = 0.3979, *p* = 0.008). Canonical correspondences analysis (CCA) was applied to reveal the influences of soil factors on the AMF community. The CCA reflected the overall correlations between AMF community and soil properties among the habitats. The total N (TN), soil organic matter (SOM), pH value, available P (AP), total P (TP) and available K (AK) had great influences on the fungal community. In the CC, the composition of AMF communities positively correlated with TN, SOM, pH and AP, and negatively correlated with TP and AK (Fig. 3). The composition of AMF communities showed opposite correlations with soil properties in the CC and CG. Meanwhile, the results of both PCA and CCA displayed no regular trend in the shift of AMF communities and the correlations between soil properties and AMF communities between different soil depths.

## Discussion

As the canopy gaps promoted the coexistence and development of plants (Dang et al., 2018), we expected that the richness and diversity of AMF in CG would be higher than that in the CC according to the ecological niche theory. Our results showed lower richness and diversity in CG, which does not support expectation (1) that the richness and diversity of AMF will be enhanced with the canopy gaps formation. The results supported our expectation (2) that the soil AMF community composition would differ notably with the generation of canopy gaps. The vegetation and soil properties are the main factors affecting AMF communities. Our result provides a theoretical background for developing new sustainable forest management, considering that selective cutting as a common sustainable forest management will also generate gaps.

Soil physicochemical properties can affect the growth and regeneration of both plants and soil microorganisms directly or indirectly (Hazard & Johnson, 2018). Ecologists have previously revealed that the soil in the canopy gaps holds more organic matter, most of which was contributed by plant wreckage (Dang et al., 2018). However, our research indicated that the SOM content in the CG was lower than in the CC. The most likely reason is that plant residues were transported away when the canopy gaps were formed in 2012 and the plant litter remained in the closed canopy. The significantly higher content of available potassium (K) in the upper soil of CG was probably caused by the characteristics of the plant leaves. gaps stimulated the growth of bush (almost broad leaves) and herb (Sabo et al., 2019). Meanwhile, K accounts for 2.5% of broad leaves dry matter compared with only 1% in coniferous leaves (Zhao, 1991).

There is a close connection between soil microorganism diversity and the vegetation on the ground, in terms of the differences in biochemical composition and nutrient content in plant litter (Russo et al., 2012; Smith, Facelli & Cavagnaro, 2018). An environment with a high diversity of plants was often accompanied by high AMF diversity (Li et al., 2010). Gerz et al. (2016) revealed that arbuscular mycorrhization was positively associated with plant species richness in a forest ecosystem. The canopy gaps transformed the plant community directly and then altered the diversity of AMF. Our results showed that both the richness and diversity of AMF in CG were lower than that in CC, which means that the AMF in CC are not only richer but also more homogeneous in the abundance of each OTU. The plant diversity hypothesis, which states that the increase of plant diversity can provide a greater variety of ecological niches resulting in a more complex soil microclimate and habitat (e.g., soil structure, root architecture), is an important theory to explain the distribution pattern of microbial diversity (Waldrop et al., 2006). However, Revillini et al. (2019) found that the diversity of AMF did not increase with the diversity of plants. In our study, contrasting to the almost bare understory under the closed canopy (2 species, described in ‘Material and Methods’), the canopy gaps nurtured more herbage and saplings (over 9 species, i.e., higher diversity). Then our results also seem to be inconsistent with the plant diversity hypothesis. Nevertheless, similar results were reported in forest, that is canopy gaps enhanced the richness of graminoids and forbs, and reduced the richness of ectomycorrhizal fungi (De Groot et al., 2016). Natural canopy gaps can also reduce the ectomycorrhizal fungi (EMF) mat concentrations in coniferous forests (Griffiths, Gray & Spies, 2010). Lewandowski et al. (2015) assessed the soil microbe community in a US northern hardwood forest using PLFA (phospholipid fatty acid) and found that the abundance of AMF initially decreased following harvest and recovered in part as time went on. We can speculate that the AMF communities were damaged due to the fragmentation of the gaps, although they could subsequently recover over time.

The AMF is obligatory biotrophic fungi and they rely on plants to survive because they cannot carry out photosynthesis (Smith & Read, 2008). The reduction of carbohydrates allocated into the below-ground of plants may be the main reason for the change of AMF community. The roots biomass of Japanese cedar in closed canopy is higher than that of the undershrub and saplings in the canopy gaps, so the cedars can provide more carbon resources for AMF. Therefore, the AMF in CC could receive enough resources, which resulted in their higher richness and diversity. In addition, plant diversity also enhanced AMF biomass by increasing C transferred to them (Mellado-Vázquez et al., 2016). Johnson & Bronstein (2019) extended the competitive exclusion principle to mutualism: the species that persists on the lowest commodity availability excludes all other competitors. Facing insufficient carbon resources, some non-dominant populations fade away, which is followed by a decreased level of diversity (Liu et al., 2009). That is partly confirmed by the fact that of the 20 OTUs with significant fluctuation in abundance, 15 (all were *Glomus*) decreased and only 5 (3 *Archaeospora*, 1 *Gigaspora* and 1 *Glomus*) increased (Appendix S1, Table SA1). Photosynthesis stimulated by increasing temperature and CO_2_ was beneficial for AMF colonization and diversity (Zavalloni et al., 2012). In the same way, CC possessed a higher richness and biodiversity index in the deeper soil layer, but CG presented the opposite trend; although they were not significant in both habitats. This trend was probably caused by the distribution of roots: Japanese cedar is an arbor with deeper roots, but most of the plants in the gaps were shrubs or saplings with shallow roots. AMF must rely on plant roots, so they presented a contrasting trend in different depths. The high percentage of the OTUs shared between habitats is reasonable, considering the cedars still stand around the gaps though we tried to avoid the gaps edge when sampling. The resources provided by the host plant are the decisive factor for the amount of AMF sustained in the plot, and different AMF species have varied preferences for the environment.

Different AM fungal taxa have individual preferences to the environment due to their different tolerance levels; therefore, the variations of soil properties caused by the forest gaps will also shift the community composition (Van Geel et al., 2018). Glomerales was the dominant group, accounting for 98.16% of sequences in CC and 56.47% in CG. This result is consistent with previous reports (Wang et al., 2019). Of the 18 AMF species detected in tropical forest, sixteen were from Glomeraceae (Husband, Herre & Young, 2002). The high-throughput sequencing results of Ma et al. (2018) also indicated that Glomerales was the dominant taxon. The main reason is that the species of Glomus survive and propagate more easily because of the ability to colonize plant roots via pieces of mycelium or mycorrhizal root fragments (Biermann & Linderman, 1983). In addition, the stronger adaptability and resistance to environmental stress of Glomus also have certain contributions to their high abundance (Öpik et al., 2009). The high relative abundance of Acaulospora in the deeper soil of CC and topsoil of CG corresponded to the low AP content. Other researchers also obtained the same results, showing that Acaulospora taxa were more effective in P uptake and transfer to the host plant compared to Glomus species (Jakobsen, Abbott & Robson, 1992). Although high throughput DNA sequencing gave us an understanding of AMF community between CG and CC in a high resolution, this result is limited due to the shortage of sample replications. Therefore, the extrapolation of our results must be very careful.

## Conclusions

Upon analysis of the results, we accept our hypothesis that canopy gaps will sharply alter the AMF community and its diversity in the *C*. *japonica* pure forest on Lushan Mountain. This is, to our knowledge, the first report describing the AM fungi community in canopy gaps using 454 pyrosequencing. Our work showed that there are abundant AM fungi in the subtropical coniferous forest despite the canopy gaps. Both the richness and diversity of AMF dropped significantly in CG, which was inconsistent with the plant diversity hypothesis (Waldrop et al., 2006). The main reason is that the shrubs or saplings in the gaps can not provide as many carbohydrates to AM fungi as the trees do (Van Geel et al., 2018). The research supported the plant investment hypothesis (Treseder, 2004) and stressed the role of plants in the symbioses with AMF (passenger hypothesis) (Horn et al., 2017). Based on the results, we recommend that removing individual trees can sustain more AMF than removing small groups of trees, at least in *C. japonica* forest. The significant increase of the relative abundance of *Archaeospora* in CG suggested that some species of *Archaeospora* may have great potential in afforestation, though further study is needed to confirm the specific species and apply them to forest management. The richness and diversity of AMF decreased with the canopy gaps formation, and additional studies are needed to explore the effects of reducing AMF diversity on forest regeneration. In the context of global climate change, global warming and increased carbon dioxide concentrations enhanced plant photosynthesis, the symbioses between AMF and plants is critical in predicting ecosystem response to global change (Kivlin, Emery & Rudgers, 2013).

##  Supplemental Information

10.7717/peerj.10905/supp-1 Data S1 Raw sequencing data

10.7717/peerj.10905/supp-2 Appendix S1 The numbers and fluctuation of OTUs in closed canopy (CC) and CG (canopy gaps)

## Additional Information and Declarations

### Data Availability

The following information was supplied regarding data availability: Data is available at NCBI BioProject accession number PRJNA656348 (SAMN15778803, SAMN15778804, SAMN15778805, SAMN15778806, SAMN15778807, SAMN15778808, SAMN15778809, SAMN157788010, SAMN157788011, SAMN157788012, SAMN157788013, SAMN157788014).
