# Supplementary material for: Depression of the soil arbuscular mycorrhizal fungal community by the canopy gaps in a Japanese cedar (Cryptomeria japonica) plantation on Lushan Mountain, subtropical China
Source: PeerJ. 2021 Mar 15;9:e10905. doi: 10.7717/peerj.10905 (PMC7971093; doi:10.7717/peerj.10905)
Supplement: Appendix S1 [file peerj-09-10905-s002.docx]

Table A1. The numbers and fluctuation of OTUs in closed canopy (CC) and CG (canopy gaps).

Yes and no mean the presence or not of the OTU, respectively; NA means the OTU only occurred in one treatment; ns means the fluctuation of the abundance of the OTU is no significant (*p* > 0.05) between CC and CG; * means the fluctuation of the abundance of the OTU is significant (*p* < 0.05), ** means very significant (*p* < 0.01); + means the abundance of the OTU increased in CG compared to CC, and – means decreased.

| **OTUID** | **Virtual taxa (in MaarjAM)** | **CC** | **CG** | **Fluctuation of abundance** |
| --- | --- | --- | --- | --- |
| OTU1 | Glomeraceae Glomus sp. VTX00126 | yes | yes | ns |
| OTU2 | Glomeraceae Glomus sp. VTX00126 | yes | yes | ns |
| OTU3 | Glomeraceae Glomus D3-Glom | yes | no | NA |
| OTU4 | Glomeraceae Glomus sp. VTX00191 | yes | yes | ns |
| OTU5 | Glomeraceae Glomus Kohout14 A-1 | yes | yes | ns |
| OTU6 | Glomeraceae Glomus sp. VTX00084 | yes | yes | **(-) |
| OTU7 | Glomeraceae Glomus Kottke08-17 VTX00124 | yes | yes | ns |
| OTU8 | Glomeraceae Glomus sp. VTX00084 | yes | yes | ns |
| OTU9 | Archaeosporaceae Archaeospora Hernandez-Hernandez17 Geo1 | yes | yes | *(+) |
| OTU10 | Glomeraceae Glomus Kottke08-17 VTX00124 | yes | yes | ns |
| OTU11 | Glomeraceae Glomus sp. VTX00291 | yes | yes | **(-) |
| OTU12 | Glomeraceae Glomus sp. VTX00080 | yes | yes | *(-) |
| OTU13 | Glomeraceae Glomus Kluber12 OTU8 VTX00074 | yes | yes | *(-) |
| OTU14 | Glomeraceae Glomus sp. | yes | yes | ns |
| OTU15 | Glomeraceae Glomus sp. VTX00080 | yes | yes | *(-) |
| OTU16 | Glomeraceae Glomus Burmannia hexaptera symbiont VTX00180 | yes | yes | ns |
| OTU17 | Glomeraceae Glomus sp. VTX00223 | yes | no | NA |
| OTU18 | Glomeraceae Glomus sp. VTX00178 | yes | no | NA |
| OTU19 | Acaulosporaceae Acaulospora sp. VTX00231 | yes | yes | ns |
| OTU20 | Glomeraceae Glomus Desiro13a MIB 8870 VTX00191 | yes | yes | ns |
| OTU21 | Glomeraceae Glomus sp. VTX00122 | yes | yes | ns |
| OTU22 | Glomeraceae Glomus LER02 VTX00219 | yes | yes | *(-) |
| OTU23 | Glomeraceae Glomus Alguacil14b Glo7 VTX00166 | yes | yes | ns |
| OTU24 | Paraglomeraceae Paraglomus IH1 VTX00444 | yes | yes | ns |
| OTU25 | Archaeosporaceae Archaeospora D5-Glom | no | yes | NA |
| OTU26 | Glomeraceae Glomus sp. VTX00080 | yes | yes | *(-) |
| OTU27 | Glomeraceae Glomus sp. VTX00126 | yes | yes | ns |
| OTU28 | Glomeraceae Glomus Kottke08-17 VTX00124 | yes | yes | ns |
| OTU29 | Glomeraceae Glomus sp. VTX00084 | yes | yes | ns |
| OTU30 | Glomeraceae Glomus sp. VTX00224 | yes | yes | *(-) |
| OTU31 | Glomeraceae Glomus sp. VTX00126 | yes | yes | ns |
| OTU32 | Glomeraceae Glomus Desiro13a MIB 8870 VTX00191 | yes | yes | ns |
| OTU33 | Glomeraceae Glomus Burmannia hexaptera symbiont VTX00180 | yes | yes | ns |
| OTU34 | Glomeraceae Glomus sp. | yes | yes | ns |
| OTU35 | Glomeraceae Glomus sp. VTX00126 | yes | yes | ns |
| OTU36 | Glomeraceae Glomus sp. VTX00080 | yes | yes | ns |
| OTU37 | Glomeraceae Glomus sp. VTX00080 | yes | yes | ns |
| OTU38 | Glomeraceae Glomus sp. VTX00088 | yes | no | NA |
| OTU39 | Glomeraceae Glomus sp. VTX00191 | yes | yes | ns |
| OTU40 | Archaeosporaceae Archaeospora D5-Glom | yes | yes | **(+) |
| OTU41 | Glomeraceae Glomus sp. VTX00080 | yes | yes | ns |
| OTU42 | Glomeraceae Glomus sp. VTX00103 | yes | yes | **(+) |
| OTU43 | Glomeraceae Glomus sp. VTX00126 | yes | yes | ns |
| OTU44 | Glomeraceae Glomus sp. VTX00080 | yes | yes | ns |
| OTU45 | Archaeosporaceae Archaeospora sp. VTX00004 | yes | yes | ns |
| OTU46 | Glomeraceae Glomus Afrothismia winkleri symbiont VTX00092 | yes | yes | ns |
| OTU47 | Glomeraceae Glomus sp. VTX00080 | yes | yes | ns |
| OTU48 | Glomeraceae Glomus Kluber12 OTU5 VTX00194 | yes | yes | ns |
| OTU49 | Glomeraceae Glomus sp. VTX00124 | yes | yes | ns |
| OTU50 | Glomeraceae Glomus sp. VTX00080 | yes | yes | ns |
| OTU51 | Glomeraceae Glomus sp. VTX00084 | yes | yes | **(-) |
| OTU52 | Glomeraceae Glomus sp. VTX00124 | yes | yes | ns |
| OTU53 | Glomeraceae Glomus sp. | no | yes | NA |
| OTU54 | Glomeraceae Glomus sp. VTX00084 | yes | yes | ns |
| OTU55 | Glomeraceae Glomus MO-G50 VTX00370 | yes | yes | ns |
| OTU56 | Archaeosporaceae Archaeospora Yoshimura13b Arch2 | yes | yes | ns |
| OTU57 | Glomeraceae Glomus sp. VTX00191 | yes | yes | ns |
| OTU58 | Glomeraceae Glomus Kluber12 OTU3 VTX00219 | yes | yes | *(-) |
| OTU59 | Glomeraceae Glomus sp. VTX00191 | yes | yes | ns |
| OTU60 | Gigasporaceae Gigaspora rosea VTX00039 | yes | yes | *(+) |
| OTU61 | Acaulosporaceae Acaulospora sp. VTX00231 | yes | yes | ns |
| OTU62 | Acaulosporaceae Acaulospora sp. VTX00012 | yes | yes | ns |
| OTU63 | Glomeraceae Glomus sp. VTX00166 | yes | yes | ns |
| OTU64 | Glomeraceae Glomus sp. VTX00219 | yes | yes | ns |
| OTU65 | Glomeraceae Glomus sp. VTX00084 | yes | yes | *(-) |
| OTU66 | Acaulosporaceae Acaulospora sp. VTX00024 | no | yes | NA |
| OTU67 | Glomeraceae Glomus sp. VTX00178 | yes | yes | ns |
| OTU68 | Glomeraceae Glomus sp. VTX00084 | yes | yes | *(-) |
| OTU69 | Glomeraceae Glomus Hernandez-Hernandez17 Rh1 | yes | no | NA |
| OTU70 | Glomeraceae Glomus Burmannia hexaptera symbiont VTX00181 | yes | yes | ns |
| OTU71 | Glomeraceae Glomus sp. VTX00126 | yes | yes | ns |
| OTU72 | Archaeosporaceae Archaeospora D5-Glom | no | yes | **(+) |
| OTU73 | Glomeraceae Glomus sp. VTX00369 | no | yes | ns |
| OTU74 | Glomeraceae Glomus Hernandez-Hernandez17 Rh2 | yes | yes | ns |
| OTU75 | Archaeosporaceae Archaeospora sp. VTX00009 | yes | no | NA |
| OTU76 | Glomeraceae Glomus sp. VTX00070 | yes | no | NA |
| OTU77 | Glomeraceae Glomus sp. | yes | yes | ns |
| OTU78 | Glomeraceae Glomus LER02 VTX00219 | yes | yes | *(-) |
| OTU79 | Archaeosporaceae Archaeospora D5-Glom | yes | yes | **(+) |
| OTU80 | Glomeraceae Glomus sp. VTX00089 | yes | yes | ns |
| OTU81 | Glomeraceae Glomus sp. VTX00166 | yes | yes | ns |
| OTU82 | Acaulosporaceae Acaulospora sp. VTX00231 | yes | yes | ns |
| OTU83 | Glomeraceae Glomus sp. VTX00383 | yes | yes | ns |
| OTU84 | Glomeraceae Glomus sp. VTX00080 | yes | yes | ns |
| OTU85 | Glomeraceae Glomus sp. VTX00080 | yes | yes | ns |
| OTU86 | Glomeraceae Glomus sp. VTX00084 | yes | yes | **(-) |
| OTU87 | Glomeraceae Glomus sp. VTX00219 | yes | no | NA |
| OTU88 | Glomeraceae Glomus sp. VTX00084 | yes | yes | **(-) |
| OTU89 | Glomeraceae Glomus sp. VTX00124 | yes | yes | ns |
| OTU90 | Glomeraceae Glomus Desiro13a MIB 8870 VTX00191 | yes | yes | ns |
| OTU91 | Acaulosporaceae Acaulospora sp. VTX00231 | yes | yes | ns |
